# Supplementary material for: Clinical Performance of the Consensus Immunoscore in Colon Cancer in the Asian Population from the Multicenter International SITC Study
Source: Cancers (Basel). 2022 Sep 6;14(18):4346. doi: 10.3390/cancers14184346 (PMC9497086; doi:10.3390/cancers14184346)
Supplement: Supplementary file 1 [file cancers-14-04346-s001.zip › cancers-1861185-supplementary.pdf]

Table S1: Demographic distribution

	Cohort1 STAGE I-III (N=423)	SITC Cohort STAGE I-III (N=2681)	Pvalue Fisher's exact test
**Age at surgery**			
N	423 (100%)	2681 (100%)	
Mean (SD)	64.7 (12.1)	67.6 (12.5)	
Range	(22-88)	(22-88)	
**Center**			
AHM (India)	58 (13.7%)	-	
SAP (Japan)	136 (32.2%)	-	
TOK (Japan)	194 (45.9%)	-	
XIA (China)	35 (8.3%)	-	
**Gender**			0,2494
Male	231 (54.6%)	1380 (51.5%)	
Female	192 (45.4%)	1301 (48.5%)	
**Age grouped**			0,0005
<65	200 (47.3%)	1024 (38.2%)	
>=65	223 (52.7%)	1657 (61.8%)	
**T Stage**			0,6214
T1	20 (4.7%)	122 (4.6%)	
T2	53 (12.5%)	402 (15.0%)	
T3	289 (68.3%)	1776 (66.2%)	
T4	61 (14.4%)	381 (14.2%)	
**N Stage**			0,1666
N0	318 (75.2%)	1885 (70.3%)	
N1	66 (15.6%)	513 (19.1%)	
N2	39 (9.2%)	250 (9.3%)	
Not Available	0 (0.0%)	33 (1.2%)	
**Number of Lymph Nodes Examined All AJCC/UICC-TNM Stages**			
N	423 (100%)	2657 (99.3%)	
Mean (SD)	16.3 (9.9)	16.8 (13.9)	
Range	(1-68)	(1-145)	
Not Available	0 (0.0%)	19 (0.7%)	
**Number of Lymph Nodes Examined AJCC/UICC-TNM Stage II only**			
N	251 (100%)	1430 (99.7%)	
Mean (SD)	65.3 (11.1)	20.1 (14.4)	
Range	(32-88)	(1-145)	
Not Available	0 (0.0%)	4 (0.3%)	
**Number of Positive Lymph Nodes AJCC/UICC-TNM Stage III only**			
N	105 (100%)	763 (100%)	
Mean (SD)	3.4 (3.5)	18.6 (14.3)	
Range	(1-27)	(1-117)	
**M Stage TNM8**			
M0	423 (100%)	2681 (100%)	
**AJCC/UICC-TNM Stage**			0,1301
I	67 (15.8%)	451 (16.8%)	
II	251 (59.3%)	1434 (53.5%)	
III	105 (24.8%)	763 (28.5%)	
Not Available	0 (0.0%)	33 (1.2%)	
**Differentiation Grade**			0,0024
Well	118 (27.9%)	601 (22.4%)	
Moderate	261 (61.7%)	1299 (48.5%)	
Poor	39 (9.2%)	322 (12.0%)	
Not Available	5 (1.2%)	459 (17.1%)	
**Proximal vs. Distal Primary (Tumor)**			0,0233
Proximal	184 (43.5%)	1340 (50.0%)	
Distal	232 (54.8%)	1322 (49.3%)	
Not Available	7 (1.7%)	19 (0.7%)	
**VELIPI**			1,0000
No	122 (28.8%)	367 (45.7%)	
Yes	301 (71.2%)	902 (33.6%)	
Not Available	0 (0.0%)	556 (20.7%)	
**Mucinous colloid type**			<.0001
No	367 (86.8%)	1962 (73.2%)	
Yes	18 (4.3%)	366 (13.7%)	
Not Available	38 (9%)	353 (13.2%)	
**MSI Status (Derived)**			<.0001
pMMR	246 (58.2%)	1275 (47.6%)	
dMMR	26 (6.1%)	304 (11.3%)	
Not Available	151 (35.7%)	1102 (41.1%)	
**Post-Operative Chemotherapy**			<.0001
No	146 (34.5%)	1856 (70%)	
Yes	265 (62.7%)	742 (28%)	
Not Available	12 (2.8%)	53 (2%)	

MSI: deficient Mismatch repair (dMMR), MSS: proficient Mismatch repair (pMMR).

Table S2: STAGE I-III Bivariable analysis for Clinical Parameters for OS and DFS

Overall Survival (OS)

	Number of patients (%)	Rate at 3 yr % (95% CI)	Rate at 5 yr % (95% CI)	Unadjusted HR (95% CI)	Unadjusted P value*	C-index (95% CI)	RMST Rel. Months (95% CI)	RMST P value**
**Age at surgery (5 groups)**						0.56 (0.49−0.64)		
<60	134 (31.7)	87 (81.3-93.2)	84.3 (78.1-91.1)	1.0 (reference)			0.0 (reference)	
>=60-70	134 (31.7)	93.2 (89.1-97.6)	88.6 (83.3-94.2)	0.73 (0.38−1.39)	0,3329		3.5 (-2.2-9.2)	0,2320
>=70-85	149 (35.2)	93.9 (90.1-97.8)	91.8 (87.5-96.4)	0.81 (0.44−1.5)	0,5116		4.5 (-0.9-9.9)	0,0995
>85	6 (1.4)	66.7 (37.9-100)	50 (22.5-100)	4.76 (1.62−13.96)	0,0045		-18 (-40.1-4)	0,1092
**Gender**						0.53 (0.46−0.59)		
Male	231 (54.6)	91 (87.3-94.8)	87.2 (82.9-91.8)	1.0 (reference)			0.0 (reference)	
Female	192 (45.4)	91.5 (87.6-95.6)	88.7 (84.3-93.4)	0.83 (0.5−1.37)	0,4650		2.4 (-10.2-14.9)	0,7113
**T stage**						0.63 (0.57−0.69)		
T1	20 (4.7)	100 (100-100)	100 (100-100)	1.0 (reference)			0.0 (reference)	
T2	53 (12.5)	96.2 (91.1-100)	94 (87.5-100)	2.02 (0.24−17.3)	0,5206		-5.6 (-20-8.8)	0,4464
T3	289 (68.3)	92.1 (89-95.3)	89.1 (85.5-92.9)	2.95 (0.41−21.49)	0,2856		-9.3 (-21.1-2.5)	0,1218
T4	61 (14.4)	79.5 (69.7-90.5)	72 (61.2-84.7)	7.82 (1.04−58.61)	0,0454		-29.1 (-46.5--11.8)	0,0010
**N stage**						0.67 (0.61−0.74)		
N0	318 (75.2)	94.9 (92.4-97.3)	92.5 (89.6-95.5)	1.0 (reference)			0.0 (reference)	
N1	66 (15.6)	88.6 (80.9-96.9)	81.8 (72.5-92.1)	2.34 (1.23−4.47)	0,0097		-12.5 (-24.1--0.8)	0,0360
N2	39 (9.2)	64.6 (50.8-82.2)	57.8 (43.5-76.9)	6.98 (3.86−12.63)	<.0001		-41.8 (-60.4--23.3)	<.0001
**AJCC/UICC-TNM stage**						0.69 (0.63−0.74)		
I	67 (15.8)	100 (100-100)	98.3 (95.1-100)	1.0 (reference)			0.0 (reference)	
II	251 (59.3)	93.5 (90.4-96.6)	90.9 (87.4-94.6)	1.97 (0.69−5.62)	0,2038		-4.9 (-14.8-4.9)	0,3249
III	105 (24.8)	79.6 (72-88)	72.9 (64.5-82.4)	6.59 (2.32−18.72)	0,0004		-31.3 (-46.1--16.6)	<.0001
**Differentiation grade**						0.62 (0.55−0.69)		
well	118 (28.2)	98.3 (96-100)	96.5 (93.2-99.9)	1.0 (reference)			0.0 (reference)	
moderate	261 (62.4)	91.2 (87.8-94.8)	87 (82.9-91.3)	2.59 (1.21−5.53)	0,0141		-12.4 (-21.7--3.1)	0,0092
poor-undiff	39 (9.3)	67.9 (54.4-84.7)	65.1 (51.4-82.4)	7.79 (3.26−18.6)	<.0001		-43.2 (-66.3--20.2)	0,0002
**Proximal vs. Distal Primary (Tumor)**						0.66 (0.6−0.72)		
proximal	184 (44.2)	89.2 (84.7-93.9)	86.8 (81.9-92)	1.0 (reference)			0.0 (reference)	
distal	232 (55.8)	93 (89.7-96.4)	88.8 (84.8-93.1)	1.05 (0.63−1.76)	0,8379		2.1 (-10.5-14.8)	0,7403
**VELIPI**						0.53 (0.47−0.58)		
NO	122 (28.8)	94 (89.7-98.4)	90 (84.5-95.8)	1.0 (reference)			0.0 (reference)	
YES	301 (71.2)	90.2 (86.9-93.7)	87.1 (83.3-91)	1.33 (0.73−2.4)	0,3541		-7.6 (-22.2-7)	0,3055
**Mucinous colloide type**						0.51 (0.48−0.55)		
NO	367 (95.3)	92.4 (89.7-95.2)	89.7 (86.6-93)	1.0 (reference)			0.0 (reference)	
YES	18 (4.7)	87.5 (72.7-100)	87.5 (72.7-100)	1.64 (0.51−5.28)	0,4068		-9.2 (-38.1-19.7)	0,5341
**MSI Status (Derived)**						0.5 (0.45−0.55)		
MSS	246 (90.4)	94.7 (92-97.6)	91.5 (88-95)	1.0 (reference)			0.0 (reference)	
MSI-H	26 (9.6)	92.3 (82.6-100)	92.3 (82.6-100)	1.35 (0.47−3.84)	0,5772		-6.2 (-24.4-12)	0,5053
**Adjuvant Chemotherapy**						0.58 (0.52−0.63)		
NO	146 (35.5)	94.5 (90.9-98.3)	93.2 (89.1-97.3)	1.0 (reference)			0.0 (reference)	
YES	265 (64.5)	89.7 (86-93.5)	85 (80.7-89.6)	1.94 (1.07−3.54)	0,0298		-12.6 (-24.8--0.4)	0,0433
**Immunoscore Lo vs Int+Hi**						0.55 (0.49−0.62)		
Lo (0-25%)	158 (37.4)	89.6 (85-94.6)	83.6 (77.9-89.7)	1.45 (0.88−2.39)	0,1434		-7.2 (-21.6-7.2)	0,3288
Int+Hi (25-100%)	265 (62.6)	92.2 (89-95.5)	90.5 (87-94.2)	1.0 (reference)			0.0 (reference)	
**Immunoscore Lo vs Int vs Hi**						0.57 (0.51−0.64)		
Lo (0-25%)	158 (37.4)	89.6 (85-94.6)	83.6 (77.9-89.7)	3.17 (1.11−9.02)	0,0306		-20.9 (-34.3--7.6)	0,0021
Int (25-70%)	197 (46.6)	90.5 (86.5-94.8)	89.4 (85.1-93.9)	2.61 (0.92−7.42)	0,0722		-18.9 (-32.2--5.7)	0,0051
Hi (70-100%)	68 (16.1)	97 (93.1-100)	93.7 (87.9-99.9)	1.0 (reference)			0.0 (reference)	
**Immunoscore**						0.58 (0.52−0.65)		
I0 (0-10%)	71 (16.8)	85.8 (78.1-94.4)	79.9 (71-89.9)	3.63 (1.21−10.85)	0,0212		-18.6 (-26.8--10.5)	<.0001
I1 (10-25%)	87 (20.6)	92.8 (87.5-98.5)	86.7 (79.7-94.3)	2.38 (0.84−6.78)	0,1035		-12 (-18--5.9)	0,0001
I2 (25-70%)	197 (46.6)	90.5 (86.5-94.8)	89.4 (85.1-93.9)	2.32 (0.76−7.11)	0,1416		-12.5 (-16.8--8.2)	<.0001
I3 (70-95%)	62 (14.7)	96.7 (92.4-100)	93.1 (86.8-99.9)	1.0 (reference)			-5.2 (-10.3--0.1)	0,0454
I4 (95-100%)	6 (1.4)	100 (100-100)	100 (100-100)	Inf (0-Inf)	NA		0.0 (reference)	
**Immunoscore Lo vs Int+Hi and High risk (T4 and VELIPI+) vs Low risk (all others)**						0.55 (0.49−0.62)		
0-25% HighRisk	11 (2.6)	81.8 (61.9-100)	71.6 (48.8-100)	2.21 (0.68−7.24)	0,1899		-12 (-36-12)	0,3282
0-25% LowRisk	147 (34.8)	90.3 (85.5-95.2)	84.5 (78.8-90.7)	1.39 (0.83−2.35)	0,211		-4.3 (-11.6-2.9)	0,2419
25-100% HighRisk	16 (3.8)	87.5 (72.7-100)	87.5 (72.7-100)	0.98 (0.24−4.11)	0,9807		-0.7 (-18.4-17.1)	0,9420
25-100% LowRisk	249 (58.9)	92.5 (89.2-95.9)	90.7 (87.1-94.5)	1.0 (reference)			0.0 (reference)	

Disease-free survival (DFS)

	Rate at 3 yr % (95% CI)	Rate at 5 yr % (95% CI)	Unadjusted HR (95% CI)	Unadjusted P value*	C-index (95% CI)	RMST Rel. Months (95% CI)	RMST P value**
**Age at surgery (5 groups)**					0.54 (0.48−0.6)		
<60	82 (75.3-89.3)	79.9 (72.8-87.6)	1.0 (reference)			0.0 (reference)	
>=60-70	83.5 (77.3-90.2)	80.2 (73.5-87.4)	0.97 (0.56−1.67)	0,9015		1.2 (-6.4-8.7)	0,7623
>=70-85	85.4 (79.9-91.4)	84 (78.2-90.2)	0.93 (0.55−1.6)	0,8009		3.2 (-4-10.3)	0,3852
>85	66.7 (37.9-100)	50 (22.5-100)	3.58 (1.24−10.34)	0,0182		-17 (-43.1-9.2)	0,2033
**Gender**					0.51 (0.45−0.56)		
Male	83.4 (78.5-88.6)	80.8 (75.6-86.4)	1.0 (reference)			0.0 (reference)	
Female	83.5 (78.3-89.1)	81.2 (75.7-87.1)	0.93 (0.6−1.42)	0,7252		1 (-14-16)	0,8988
**T stage**					0.62 (0.57−0.67)		
T1	100 (100-100)	100 (100-100)	1.0 (reference)			0.0 (reference)	
T2	93.9 (87.5-100)	91.5 (83.8-99.9)	2.65 (0.32−22)	0,3672		-9.7 (-25.6-6.1)	0,2297
T3	83.8 (79.4-88.3)	81.3 (76.7-86.2)	4.81 (0.67−34.78)	0,1193		-19.3 (-31.7--6.9)	0,0023
T4	67.6 (56.5-80.7)	63.6 (52.3-77.4)	10.18 (1.37−75.54)	0,0233		-40.4 (-59.3--21.6)	<.0001
**N stage**					0.66 (0.61−0.72)		
N0	89.6 (86.2-93.1)	87.8 (84.1-91.6)	1.0 (reference)			0.0 (reference)	
N1	78.6 (68.8-89.6)	75 (64.7-86.9)	2.03 (1.15−3.59)	0,0152		-14.5 (-28.5--0.4)	0,0437
N2	35.9 (22.4-57.7)	28.7 (16.3-50.6)	7.91 (4.77−13.13)	<.0001		-66.1 (-84.2--48)	<.0001
**AJCC/UICC-TNM stage**					0.68 (0.63−0.73)		
I	98.4 (95.4-100)	96.6 (92.1-100)	1.0 (reference)			0.0 (reference)	
II	87.2 (83.1-91.6)	85.4 (81-90.1)	2.33 (0.92−5.89)	0,0753		-10.6 (-22.4-1.3)	0,0797
III	63.8 (54.7-74.4)	59 (49.7-70.1)	7.34 (2.89−18.64)	<.0001		-46.2 (-63.2--29.2)	<.0001
**Differentiation grade**					0.62 (0.57−0.67)		
well	91.4 (86.5-96.6)	90.5 (85.3-96)	1.0 (reference)			0.0 (reference)	
moderate	82.6 (77.9-87.6)	79.9 (74.9-85.2)	2.03 (1.15−3.61)	0,0153		-16.1 (-28.1--4.1)	0,0084
poor-undiff	63.2 (49-81.3)	56.1 (41.5-75.9)	4.76 (2.32−9.75)	<.0001		-44.4 (-70.1--18.8)	0,0007
**Proximal vs. Distal Primary (Tumor)**					0.51 (0.45−0.56)		
proximal	82 (76.3-88)	80 (74.2-86.4)	1.0 (reference)			0.0 (reference)	
distal	84.5 (79.9-89.5)	81.6 (76.6-86.9)	0.98 (0.64−1.51)	0,9390		1.4 (-13.9-16.7)	0,8587
**VELIPI**					0.52 (0.48−0.57)		
NO	88.1 (81.9-94.7)	84.5 (77.6-92.1)	1.0 (reference)			0.0 (reference)	
YES	81.9 (77.6-86.4)	79.7 (75.2-84.5)	1.21 (0.72−2.01)	0,4684		-5.7 (-24.4-13)	0,5487
**Mucinous colloide type**					0.51 (0.48−0.54)		
NO	84.9 (81.2-88.7)	83.3 (79.5-87.3)	1.0 (reference)			0.0 (reference)	
YES	75.1 (56.6-99.7)	75.1 (56.6-99.7)	1.37 (0.5−3.76)	0,5404		-7.7 (-39.7-24.2)	0,6352
**MSI Status (Derived)**					0.52 (0.48−0.55)		
MSS	86.2 (82-90.6)	84.5 (80.1-89.2)	1.0 (reference)			0.0 (reference)	
MSI-H	92.3 (82.6-100)	92.3 (82.6-100)	0.87 (0.31−2.42)	0,7855		1.3 (-18.4-21)	0,8974
**Adjuvant Chemotherapy**					0.57 (0.51−0.63)		
NO	89.4 (84.5-94.6)	89.4 (84.5-94.6)	1.0 (reference)			0.0 (reference)	
YES	80.4 (75.6-85.6)	76.3 (71.1-82)	1.91 (1.15−3.17)	0,0119		-17.4 (-32.3--2.4)	0,0225
**Immunoscore Lo vs Int+Hi**					0.55 (0.5−0.61)		
Lo (0-25%)	78.2 (71.9-85.1)	75.4 (68.8-82.6)	1.47 (0.96−2.25)	0,0776		-11.8 (-29-5.4)	0,1798
Int+Hi (25-100%)	86.8 (82.6-91.2)	84.5 (80-89.2)	1.0 (reference)			0.0 (reference)	
**Immunoscore Lo vs Int vs Hi**					0.58 (0.53−0.63)		
Lo (0-25%)	78.2 (71.9-85.1)	75.4 (68.8-82.6)	4.13 (1.48−11.54)	0,0069		-34.4 (-50.3--18.6)	<.0001
Int (25-70%)	83.7 (78.5-89.2)	81.9 (76.5-87.7)	3.42 (1.22−9.54)	0,0190		-30 (-45.2--14.7)	0,0001
Hi (70-100%)	96.6 (92.2-100)	92.8 (86.2-99.9)	1.0 (reference)			0.0 (reference)	
**Immunoscore**					0.59 (0.53−0.64)		
I0 (0-10%)	74 (64.3-85.1)	72.3 (62.5-83.8)	4.36 (1.49−12.76)	0,0072		-17 (-23.6--10.3)	<.0001
I1 (10-25%)	81.8 (73.8-90.6)	78 (69.5-87.5)	3.52 (1.2−10.31)	0,0215		-13.1 (-18.6--7.7)	<.0001
I2 (25-70%)	83.7 (78.5-89.2)	81.9 (76.5-87.7)	3.23 (1.16−9.01)	0,0254		-11.5 (-15.1--8)	<.0001
I3 (70-95%)	96.4 (91.6-100)	92.3 (85.3-99.9)	1.0 (reference)			-3.4 (-7-0.2)	0,0659
I4 (95-100%)	100 (100-100)	100 (100-100)	Inf (0-Inf)	NA		0.0 (reference)	
**Immunoscore Lo vs Int+Hi and High risk (T4 and VELIPI+) vs Low risk (all others)**					0.56 (0.5−0.62)		
0-25% HighRisk	63.6 (40.7-99.5)	63.6 (40.7-99.5)	2.32 (0.83−6.46)	0,1083		-21.2 (-53.6-11.3)	0,2009
0-25% LowRisk	79.4 (72.9-86.3)	76.3 (69.6-83.7)	1.37 (0.88−2.14)	0,1597		-6.8 (-16-2.4)	0,1485
25-100% HighRisk	87.5 (72.7-100)	87.5 (72.7-100)	0.64 (0.16−2.65)	0,5409		5.5 (-13.6-24.7)	0,5707
25-100% LowRisk	86.7 (82.4-91.3)	84.3 (79.6-89.2)	1.0 (reference)			0.0 (reference)	

* Wald P Value. ** Restricted Mean Survival Time (RMST) P value. MSS: proficient Mismatch repair (pMMR)

Table S3: STAGE I-III MSS and STAGE II Bivariable analysis for IS Parameters for TTR, OS and DFS

Time To Recurrence (TTR)

	Number of patients (%)	Rate at 3 yr % (95% CI)	Rate at 5 yr % (95% CI)	Unadjusted HR (95% CI)	P value*	C-index (95% CI)	RMST Rel. Months (95% CI)	P value**
**STAGE I-III MSS**								
**Immunoscore Lo vs Int+Hi**						0.68 (0.61−0.75)		
Lo (0-25%)	94 (38.2)	73.4 (65-82.9)	71.2 (62.7-81)	4.58 (2.27−9.23)	<.0001		-39.6 (-57.8--21.4)	<.0001
Int+Hi (25-100%)	152 (61.8)	94.1 (90.4-97.9)	92.8 (88.7-97)	1.0 (reference)			0.0 (reference)	
**Immunoscore Lo vs Int vs Hi**						0.7 (0.63−0.76)		
Lo (0-25%)	94 (38.2)	73.4 (65-82.9)	71.2 (62.7-81)	10.93 (1.49−80.49)	0,0188		-43.6 (-60.6--26.6)	<.0001
Int (25-70%)	120 (48.8)	92.5 (87.9-97.3)	91.7 (86.9-96.7)	2.77 (0.36−21.67)	0,3307		-9.6 (-20.6-1.5)	0,0892
Hi (70-100%)	32 (13)	96.9 (91-100)	96.9 (91-100)	1.0 (reference)			0.0 (reference)	
**Immunoscore**						0.71 (0.63−0.78)		
I0 (0-10%)	39 (15.9)	69.2 (56.2-85.3)	66.6 (53.3-83.2)	12.85 (1.68−98.28)	0,0139		-19.9 (-29--10.7)	<.0001
I1 (10-25%)	55 (22.4)	76.4 (65.9-88.5)	74.5 (63.9-87)	9.11 (1.2−69.3)	0,0328		-14.8 (-21.7--7.9)	<.0001
I2 (25-70%)	120 (48.8)	92.5 (87.9-97.3)	91.7 (86.9-96.7)	2.69 (0.34−20.99)	0,3460		-4.6 (-7.4--1.8)	0,0013
I3 (70-95%)	31 (12.6)	96.8 (90.8-100)	96.8 (90.8-100)	1.0 (reference)			-0.6 (-1.8-0.6)	0,3094
I4 (95-100%)	1 (0.4)	100 (100-100)	100 (100-100)	Inf (0−Inf)	NA		0.0 (reference)	
**STAGE I-III MSI-H**								
**Immunoscore Lo vs Int+Hi**						0.6 (0.52-0.68)		
Lo (0-25%)	5 (19.2)	100 (100-100)	100 (100-100)	NA (NA-NA)	0,4848		10.3 (-3.3-24)	0,1372
Int+Hi (25-100%)	21 (80.8)	90.5 (78.8-100)	90.5 (78.8-100)	1.0 (reference)			0.0 (reference)	
**STAGE II**								
**Immunoscore Lo vs Int+Hi**						0.62 (0.54−0.7)		
Lo (0-25%)	102 (40.6)	80.4 (72.9-88.7)	78.3 (70.4-86.9)	2.72 (1.35−5.51)	0,0052		-25.3 (-42.8--7.8)	0,0046
Int+Hi (25-100%)	149 (59.4)	92.7 (88.4-97.2)	91.1 (86.4-96.1)	1.0 (reference)			0.0 (reference)	
**Immunoscore Lo vs Int vs Hi**						0.63 (0.55−0.71)		
Lo (0-25%)	102 (40.6)	80.4 (72.9-88.7)	78.3 (70.4-86.9)	3.82 (0.9−16.24)	0,0697		-27.7 (-46.8--8.7)	0,0043
Int (25-70%)	112 (44.6)	91.5 (86.3-97)	90.5 (85-96.3)	1.52 (0.33−6.95)	0,5867		-6.2 (-22.3-9.9)	0,4526
Hi (70-100%)	37 (14.7)	96.9 (91-100)	93.5 (85.2-100)	1.0 (reference)			0.0 (reference)	
**Immunoscore**						0.63 (0.55−0.72)		
I0 (0-10%)	47 (18.7)	78.2 (67.1-91.1)	75.8 (64.3-89.4)	3.89 (0.86−17.57)	0,0771		-13.7 (-20.9--6.4)	0,0002
I1 (10-25%)	55 (21.9)	82.5 (72.7-93.6)	80.4 (70.3-92.1)	3.45 (0.76−15.56)	0,1074		-11.8 (-18.4--5.2)	0,0005
I2 (25-70%)	112 (44.6)	91.5 (86.3-97)	90.5 (85-96.3)	1.46 (0.32−6.66)	0,6256		-5.7 (-9.2--2.2)	0,0013
I3 (70-95%)	34 (13.5)	96.7 (90.5-100)	93.2 (84.6-100)	1.0 (reference)			-3 (-7.5-1.6)	0,2050
I4 (95-100%)	3 (1.2)	100 (100-100)	100 (100-100)	Inf (0−Inf)	NA		0.0 (reference)	
**STAGE-II Very High Risk (T4 and VELIPI+) and IS**						0.54 (0.47−0.61)		
Very-High-Risk 0-25%	11 (4.4)	63.6 (40.7-99.5)	63.6 (40.7-99.5)	3.4 (1.19−9.71)	0,0222		-26.9 (-59.2-5.5)	0,1034
Very-High-Risk 25-100%	16 (6.4)	87.5 (72.7-100)	87.5 (72.7-100)	0.94 (0.22−3.96)	0,9368		-0.2 (-19.2-18.8)	0,9845
Low-Risk	224 (89.2)	88.8 (84.6-93.2)	86.8 (82.2-91.6)	1.0 (reference)			0.0 (reference)	
**STAGE-II High Risk (T4 or VELIPI+) and IS**						0.63 (0.54−0.72)		
High-Risk 0-25%	77 (30.7)	78.6 (69.9-88.5)	75.8 (66.6-86.2)	1.79 (0.75−4.28)	0,1918		-13.9 (-33.2-5.4)	0,1572
High-Risk 25-100%	108 (43)	93.5 (88.9-98.3)	91.5 (86.4-97)	0.58 (0.22−1.56)	0,2831		8.2 (-7.3-23.7)	0,2987
Low-Risk	66 (26.3)	88.6 (80.4-97.6)	88.6 (80.4-97.6)	1.0 (reference)			0.0 (reference)	
**STAGE II Very High Risk (T4 and VELIPI+)**								
**Immunoscore Lo vs Int+Hi**						0.64 (0.44−0.84)		
Lo (0-25%)	11 (40.7)	63.6 (40.7-99.5)	63.6 (40.7-99.5)	3.17 (0.58−17.33)	0,1828		-26.7 (-63.6-10.2)	0,1560
Int+Hi (25-100%)	16 (59.3)	87.5 (72.7-100)	87.5 (72.7-100)	1.0 (reference)			0.0 (reference)	
**STAGE II Low Risk (not (T4 and VELIPI+))**								
**Immunoscore Lo vs Int+Hi**						0.61 (0.52−0.71)		
Lo (0-25%)	91 (40.6)	82.6 (75-91)	80.2 (72.2-89.1)	2.62 (1.21−5.68)	0,0146		-18 (-32.3--3.7)	0,0135
Int+Hi (25-100%)	133 (59.4)	93.4 (89-97.9)	91.6 (86.7-96.7)	1.0 (reference)			0.0 (reference)	
**STAGE II High Risk (T4 or VELIPI+)**								
**Immunoscore Lo vs Int+Hi**						0.64 (0.55−0.73)		
Lo (0-25%)	77 (41.6)	78.6 (69.9-88.5)	75.8 (66.6-86.2)	3.11 (1.39−6.91)	0,0055		-22.1 (-37.5--6.7)	0,0050
Int+Hi (25-100%)	108 (58.4)	93.5 (88.9-98.3)	91.5 (86.4-97)	1.0 (reference)			0.0 (reference)	

Overall survival (OS)

	Rate at 3 yr % (95% CI)	Rate at 5 yr % (95% CI)	Unadjusted HR (95% CI)	P value*	C-index (95% CI)	RMST Rel. Months (95% CI)	P value**
**STAGE I-III MSS**							
**Immunoscore Lo vs Int+Hi**					0.64 (0.57−0.72)		
Lo (0-25%)	87.2 (80.7-94.2)	80.9 (73.3-89.2)	5.3 (2.25−12.49)	0,0001		-28 (-44.8--11.2)	0,0011
Int+Hi (25-100%)	99.3 (98.1-100)	98 (95.8-100)	1.0 (reference)			0.0 (reference)	
**Immunoscore Lo vs Int vs Hi**					0.72 (0.64−0.8)		
Lo (0-25%)	87.2 (80.7-94.2)	80.9 (73.3-89.2)	7.67 (1.03−57.09)	0,0466		-30.3 (-45.4--15.3)	0,0001
Int (25-70%)	99.2 (97.6-100)	98.3 (96.1-100)	1.56 (0.19−12.98)	0,6794		-5.7 (-16.9-5.5)	0,3205
Hi (70-100%)	96.9 (91-100)	96.9 (91-100)	1.0 (reference)			0.0 (reference)	
**Immunoscore**					0.73 (0.64−0.82)		
I0 (0-10%)	84.6 (74-96.7)	76.9 (64.8-91.4)	8.69 (1.11−67.98)	0,0394		-11.4 (-17.8--4.9)	0,0006
I1 (10-25%)	89.1 (81.2-97.7)	83.6 (74.4-94)	6.57 (0.85−50.92)	0,0716		-7.7 (-12.3--3.1)	0,0011
I2 (25-70%)	99.2 (97.6-100)	98.3 (96.1-100)	1.51 (0.18−12.58)	0,7012		-1.1 (-2.3-0)	0,0560
I3 (70-95%)	96.8 (90.8-100)	96.8 (90.8-100)	1.0 (reference)			-0.6 (-1.8-0.6)	0,3094
I4 (95-100%)	100 (100-100)	100 (100-100)	Inf (0−Inf)	NA		0.0 (reference)	
**STAGE I-III MSI-H**							
**Immunoscore Lo vs Int+Hi**					0.61 (0.52-0.71)		
Lo (0-25%)	100 (100-100)	100 (100-100)	0 (0-Inf)	0,2633		16.9 (2.3-31.4)	0,0229
Int+Hi (25-100%)	90.5 (78.8-100)	90.5 (78.8-100)	1.0 (reference)			0.0 (reference)	
**STAGE II**							
**Immunoscore Lo vs Int+Hi**					0.57 (0.48−0.67)		
Lo (0-25%)	91.1 (85.7-96.8)	86 (79.5-93.1)	1.67 (0.79−3.5)	0,1784		-9 (-24.3-6.3)	0,2481
Int+Hi (25-100%)	95.2 (91.8-98.7)	94.5 (90.8-98.3)	1.0 (reference)			0.0 (reference)	
**Immunoscore Lo vs Int vs Hi**					0.58 (0.49−0.68)		
Lo (0-25%)	91.1 (85.7-96.8)	86 (79.5-93.1)	2.67 (0.61−11.67)	0,1926		-14.9 (-30.8-1)	0,0655
Int (25-70%)	94.5 (90.4-98.9)	94.5 (90.4-98.9)	1.8 (0.4−8.11)	0,4454		-8.9 (-24-6.2)	0,2491
Hi (70-100%)	97.2 (92-100)	94.3 (86.9-100)	1.0 (reference)			0.0 (reference)	
**Immunoscore**					0.6 (0.49−0.7)		
I0 (0-10%)	89.4 (81-98.6)	82.8 (72.6-94.4)	2.93 (0.62−13.81)	0,1738		-12 (-19.7--4.3)	0,0022
I1 (10-25%)	92.6 (85.9-99.8)	88.8 (80.8-97.7)	2.12 (0.44−10.23)	0,3475		-8.1 (-13.9--2.4)	0,0058
I2 (25-70%)	94.5 (90.4-98.9)	94.5 (90.4-98.9)	1.68 (0.37−7.57)	0,5006		-6.5 (-10.3--2.7)	0,0009
I3 (70-95%)	97 (91.3-100)	93.8 (85.9-100)	1.0 (reference)			-4.1 (-9.8-1.7)	0,1666
I4 (95-100%)	100 (100-100)	100 (100-100)	Inf (0−Inf)	NA		0.0 (reference)	
**STAGE-II Very High Risk (T4 and VELIPI+) and IS**					0.55 (0.47−0.62)		
Very-High-Risk 0-25%	81.8 (61.9-100)	71.6 (48.8-100)	2.92 (0.88−9.75)	0,0813		-15 (-39-9)	0,2204
Very-High-Risk 25-100%	87.5 (72.7-100)	87.5 (72.7-100)	1.27 (0.3−5.39)	0,7462		-3.7 (-21.3-14)	0,6846
Low-Risk	94.5 (91.5-97.6)	92.1 (88.6-95.8)	1.0 (reference)			0.0 (reference)	
**STAGE-II High Risk (T4 or VELIPI+) and IS**					0.58 (0.48−0.69)		
High-Risk 0-25%	89.6 (83-96.7)	84.2 (76.4-92.8)	2.16 (0.77−6.06)	0,1438		-11.4 (-25.7-2.8)	0,1155
High-Risk 25-100%	95.3 (91.4-99.4)	94.4 (90.1-98.9)	1.17 (0.4−3.44)	0,7706		-2.8 (-14.9-9.4)	0,6545
Low-Risk	95.2 (90.1-100)	93.5 (87.5-99.9)	1.0 (reference)			0.0 (reference)	
**STAGE II Very High Risk (T4 and VELIPI+)**							
**Immunoscore Lo vs Int+Hi**					0.58 (0.35−0.81)		
Lo (0-25%)	81.8 (61.9-100)	71.6 (48.8-100)	2.16 (0.36−12.97)	0,3990		-11.3 (-40.6-17.9)	0,4479
Int+Hi (25-100%)	87.5 (72.7-100)	87.5 (72.7-100)	1.0 (reference)			0.0 (reference)	
**STAGE II Low Risk (not (T4 and VELIPI+))**							
**Immunoscore Lo vs Int+Hi**					0.57 (0.46−0.67)		
Lo (0-25%)	92.2 (86.9-97.9)	87.7 (81.2-94.8)	1.56 (0.69−3.54)	0,2859		-6.1 (-18.1-6)	0,3246
Int+Hi (25-100%)	96.1 (92.8-99.5)	95.3 (91.7-99)	1.0 (reference)			0.0 (reference)	
**STAGE II High Risk (T4 or VELIPI+)**							
**Immunoscore Lo vs Int+Hi**					0.59 (0.49−0.69)		
Lo (0-25%)	89.6 (83-96.7)	84.2 (76.4-92.8)	1.83 (0.8−4.17)	0,1530		-8.7 (-22.4-5)	0,2152
Int+Hi (25-100%)	95.3 (91.4-99.4)	94.4 (90.1-98.9)	1.0 (reference)			0.0 (reference)	

Disease-free survival (DFS)

	Rate at 3 yr % (95% CI)	Rate at 5 yr % (95% CI)	Unadjusted HR (95% CI)	P value*	C-index (95% CI)	RMST Rel. Months (95% CI)	P value**
**STAGE I-III MSS**							
**Immunoscore Lo vs Int+Hi**					0.67 (0.6−0.74)		
Lo (0-25%)	73.4 (65-82.9)	71.2 (62.7-81)	3.69 (1.94−7.02)	<.0001		-36.4 (-56.3--16.4)	0,0004
Int+Hi (25-100%)	94.1 (90.4-97.9)	92.8 (88.7-97)	1.0 (reference)			0.0 (reference)	
**Immunoscore Lo vs Int vs Hi**					0.68 (0.62−0.75)		
Lo (0-25%)	73.4 (65-82.9)	71.2 (62.7-81)	11.1 (1.51−81.6)	0,0181		-45.8 (-63.1--28.6)	<.0001
Int (25-70%)	92.5 (87.9-97.3)	91.7 (86.9-96.7)	3.55 (0.46−27.14)	0,2222		-15.3 (-28.3--2.3)	0,0207
Hi (70-100%)	96.9 (91-100)	96.9 (91-100)	1.0 (reference)			0.0 (reference)	
**Immunoscore**					0.69 (0.62−0.77)		
I0 (0-10%)	69.2 (56.2-85.3)	66.6 (53.3-83.2)	12.47 (1.63−95.42)	0,0151		-19.9 (-29--10.7)	<.0001
I1 (10-25%)	76.4 (65.9-88.5)	74.5 (63.9-87)	9.61 (1.27−72.81)	0,0284		-14.8 (-21.7--7.9)	<.0001
I2 (25-70%)	92.5 (87.9-97.3)	91.7 (86.9-96.7)	3.44 (0.45−26.31)	0,2337		-4.9 (-7.7--2)	0,0007
I3 (70-95%)	96.8 (90.8-100)	96.8 (90.8-100)	1.0 (reference)			-0.6 (-1.8-0.6)	0,3094
I4 (95-100%)	100 (100-100)	100 (100-100)	Inf (0−Inf)	NA		0.0 (reference)	
**STAGE I-III MSI-H**							
**Immunoscore Lo vs Int+Hi**					0.61 (0.52-0.71)		
Lo (0-25%)	100 (100-100)	100 (100-100)	0 (0-Inf)	0,2633		18.5 (2.4-34.6)	0,0245
Int+Hi (25-100%)	90.5 (78.8-100)	90.5 (78.8-100)	1.0 (reference)			0.0 (reference)	
**STAGE II**							
**Immunoscore Lo vs Int+Hi**					0.59 (0.52−0.67)		
Lo (0-25%)	80.4 (72.9-88.7)	78.3 (70.4-86.9)	1.46 (0.74−2.88)	0,2711		-21 (-40.4--1.6)	0,0342
Int+Hi (25-100%)	92.1 (87.7-96.7)	90.5 (85.7-95.6)	1.0 (reference)			0.0 (reference)	
**Immunoscore Lo vs Int vs Hi**					0.61 (0.53−0.68)		
Lo (0-25%)	80.4 (72.9-88.7)	78.3 (70.4-86.9)	4.02 (0.95−17.04)	0,0594		-30.6 (-50.2--11)	0,0022
Int (25-70%)	90.7 (85.3-96.4)	89.7 (84.1-95.6)	2.25 (0.51−9.84)	0,2813		-14.7 (-32.2-2.9)	0,1009
Hi (70-100%)	96.9 (91-100)	93.5 (85.2-100)	1.0 (reference)			0.0 (reference)	
**Immunoscore**					0.61 (0.53−0.69)		
I0 (0-10%)	78.2 (67.1-91.1)	75.8 (64.3-89.4)	3.92 (0.87−17.69)	0,0756		-13.7 (-20.9--6.4)	0,0002
I1 (10-25%)	82.5 (72.7-93.6)	80.4 (70.3-92.1)	3.81 (0.85−17.01)	0,0801		-11.8 (-18.4--5.2)	0,0005
I2 (25-70%)	90.7 (85.3-96.4)	89.7 (84.1-95.6)	2.16 (0.49−9.46)	0,3054		-6.4 (-10--2.9)	0,0004
I3 (70-95%)	96.7 (90.5-100)	93.2 (84.6-100)	1.0 (reference)			-3 (-7.5-1.6)	0,2050
I4 (95-100%)	100 (100-100)	100 (100-100)	Inf (0−Inf)	NA		0.0 (reference)	
**STAGE-II Very High Risk (T4 and VELIPI+) and IS**					0.55 (0.47−0.62)		
Very-High-Risk 0-25%	63.6 (40.7-99.5)	63.6 (40.7-99.5)	2.92 (0.88−9.75)	0,0813		-24.4 (-56.8-8)	0,1397
Very-High-Risk 25-100%	87.5 (72.7-100)	87.5 (72.7-100)	1.27 (0.3−5.39)	0,7462		2.3 (-16.8-21.4)	0,8140
Low-Risk	88.4 (84.2-92.9)	86.4 (81.8-91.2)	1.0 (reference)			0.0 (reference)	
**STAGE-II High Risk (T4 or VELIPI+) and IS**					0.58 (0.48−0.69)		
High-Risk 0-25%	78.6 (69.9-88.5)	75.8 (66.6-86.2)	2.16 (0.77−6.06)	0,1438		-12.5 (-32.7-7.7)	0,2256
High-Risk 25-100%	93.5 (88.9-98.3)	91.5 (86.4-97)	1.17 (0.4−3.44)	0,7706		7.6 (-9.2-24.4)	0,3761
Low-Risk	87 (78.5-96.5)	87 (78.5-96.5)	1.0 (reference)			0.0 (reference)	
**STAGE II Very High Risk (T4 and VELIPI+)**							
**Immunoscore Lo vs Int+Hi**					0.64 (0.44−0.84)		
Lo (0-25%)	63.6 (40.7-99.5)	63.6 (40.7-99.5)	3.17 (0.58−17.33)	0,1828		-26.7 (-63.6-10.2)	0,1560
Int+Hi (25-100%)	87.5 (72.7-100)	87.5 (72.7-100)	1.0 (reference)			0.0 (reference)	
**STAGE II Low Risk (not (T4 and VELIPI+))**							
**Immunoscore Lo vs Int+Hi**					0.58 (0.5−0.67)		
Lo (0-25%)	82.6 (75-91)	80.2 (72.2-89.1)	1.88 (0.96−3.7)	0,0676		-14.4 (-30.2-1.3)	0,0727
Int+Hi (25-100%)	92.6 (88.1-97.4)	90.9 (85.9-96.2)	1.0 (reference)			0.0 (reference)	
**STAGE II High Risk (T4 or VELIPI+)**							
**Immunoscore Lo vs Int+Hi**					0.62 (0.53−0.71)		
Lo (0-25%)	78.6 (69.9-88.5)	75.8 (66.6-86.2)	2.46 (1.2−5.08)	0,0146		-20.1 (-36.9--3.3)	0,0191
Int+Hi (25-100%)	93.5 (88.9-98.3)	91.5 (86.4-97)	1.0 (reference)			0.0 (reference)	

* Wald P Value. ** Restricted Mean Survival Time (RMST) P value. MSS: proficient Mismatch repair (pMMR)

Table S4: Multivariable analysis IS vs clinical parameters for TTR, OS and DFS in Stage I-III

	TTR Model (40/266)*			OS Model (32/266)*			DFS Model (46/266)*		
	Hazard Ratio (95% CI)	P-value[1]	C-Index (95% CI)	Hazard Ratio (95% CI)	P-value[1]	C-Index (95% CI)	Hazard Ratio (95% CI)	P-value[1]	C-Index (95% CI)
**Multivariable Stratified Cox Model**			0,75 (0.68-0.81)			0,76 (0.69-0.83)			0,74 (0.67-0.81)
**Immunoscore**									
Lo *vs* Int+Hi	2.22 (1.10-4.55)	0,0269		2.44 (1.01-5.55)	0,0304		1.89 (1.00-3.57)	0,0516	
**Age at surgery**									
continuous	1.02 (0.98-1.05)	0,3567		1.04 (1-1.08)	0,0659		1.03 (0.99-1.06)	0,0967	
**Gender**									
Female *vs* Male	1.08 (0.56-2.07)	0,8273		0.85 (0.41-1.77)	0,6695		1.06 (0.57-1.97)	0,8577	
**T Stage**									
T3 *vs* T1-2	9.43 (1.26-70.64)	0,0289		6.91 (0.91-52.6)	0,0618		5.67 (1.33-24.21)	0,0191	
T4 *vs* T1-2	14.35 (1.73-119.27)	0,0137		11.03 (1.28-95.1)	0,0289		7.92 (1.6-39.16)	0,0111	
**N Stage**									
N1 *vs* N0	0.64 (0.25-1.64)	0,355		0.78 (0.28-2.14)	0,6240		0.62 (0.25-1.56)	0,3113	
N2 *vs* N0	2.35 (1.13-4.89)	0,0223		1.81 (0.72-4.59)	0,2082		2.53 (1.24-5.15)	0,0105	
**Sidedness**									
Distal *vs* proximal	0.97 (0.49-1.93)	0,9307		0.98 (0.44-2.19)	0,9689		1.1 (0.57-2.12)	0,7789	
**MSI Status (Derived)**									
dMMR *vs* pMMR	1.08 (0.24-4.81)	0,9177		2.9 (0.9-9.33)	0,0742		1.8 (0.59-5.43)	0,2994	

* (Events/Total); [1]Stratified covariate Wald p-value; Stratified by center. MSS: proficient Mismatch repair (pMMR)

Section & Topic	No Item	details	Checklist
**TITLE OR ABSTRACT**	1	Identification as a study of diagnostic accuracy using at least one measure of accuracy	Yes
**ABSTRACT**	2	Structured summary of study design, methods, results, and conclusions (for specific guidance, see STARD for Abstracts)	Yes
**INTRODUCTION**	3	Scientific and clinical background, including the intended use and clinical role of the index test	Yes (novel TNM-Immune classification for Stage I/III colon cancer)
	4	Study objectives and hypotheses	Yes
**METHODS**			
**Study design**	5	Whether data collection was planned before the index test and reference standard were performed (prospective study) or after (retrospective study)	Yes, Retrospective study using pre-defined test and scoring method and cutoff, on 3 independent datasets
**Participants**	6	Eligibility criteria	Yes, (Inclusions: Patient with colon cancer ; Age > 18 years ; Patient had surgery performed prior to 2010. (5 years follow-up is recommended) ; Therapeutic procedures (surgery, radiotherapy, chemotherapy, biotherapy, ...) and follow-up registered; T1, T2, T3, T4 tumors ; All N stages ; pM0 at the time of diagnosis ; Adjuvant chemotherapy is allowed). (Exclusion: Neo-adjuvant treatment, rectum cancer)
	7	On what basis potentially eligible participants were identified (such as symptoms, results from previous tests, inclusion in registry)	Yes, (patients with eligibility criteria from participating centers)
	8	Where and when potentially eligible participants were identified (setting, location and dates)	Yes, (from participating centers)
	9	Whether participants formed a consecutive, random or convenience series	Yes (patients with eligibility criteria from participating centers, randomly selected with 5 years follow-up)
**Test methods**	10a	Index test, in sufficient detail to allow replication	Yes (multiple reproducibility data and details fro replication)
	10b	Reference standard, in sufficient detail to allow replication	Yes (AJCC/UICC-TNM)
	11	Rationale for choosing the reference standard (if alternatives exist)	Yes, (meta-analysis for available evidence of the association of immune cell infiltrates with prognosis in various types of cancers)
	12a	Definition of and rationale for test positivity cut-offs or result categories of the index test, distinguishing pre-specified from exploratory	Yes, (Test method was pre-defined, cutoff were defined in the Training set, and subsequently validated in the independent datasets)
	12b	Definition of and rationale for test positivity cut-offs or result categories of the reference standard, distinguishing pre-specified from exploratory	Yes (AJCC/UICC-TNM)
	13a	Whether clinical information and reference standard results were available to the performers/readers of the index test	Yes, (clinical information, percentiles methods and cutoff of the test are given)
	13b	Whether clinical information and index test results were available to the assessors of the reference standard	Reference (AJCC/UICC-TNM); Test was performed blinded to clinical data and reference standard. Reference standard was done blinded to Test.
**Analysis**	14	Methods for estimating or comparing measures of diagnostic accuracy	Yes, (several methods, including Cox multivariate analyses with Logrank P-values, Wald P-values, Chi2 risk contribution, and Harrel's c-index are reported
	15	How indeterminate index test or reference standard results were handled	Yes, (patients with indeterminate data were excluded from the study)
	16	How missing data on the index test and reference standard were handled	Yes, (patients with missing data were excluded from the study)
	17	Any analyses of variability in diagnostic accuracy, distinguishing pre-specified from exploratory	Yes, (Test was pre-specified, test method was pre-defined, cutoff were defined in the Training set, and subsequently validated in the independent datasets)
	18	Intended sample size and how it was determined	Yes (>600 patients in each dataset, and >1200 Stage II, based on HR/P-values from previous studies)
**RESULTS**			
**Participants**	19	Flow of participants, using a diagram	Yes (Fig S1)
	20	Baseline demographic and clinical characteristics of participants	Yes (Table S1)
	21a	Distribution of severity of disease in those with the target condition	Yes (see appendix tables)
	21b	Distribution of alternative diagnoses in those without the target condition	na
	22	Time interval and any clinical interventions between index test and reference standard	Yes (Standard of care from real-life cancers in each center)
**Test results**	23	Cross tabulation of the index test results (or their distribution) by the results of the reference standard	Yes (see appendix tables)
	24	Estimates of diagnostic accuracy and their precision (such as 95% confidence intervals)	Yes (see appendix tables)
	25	Any adverse events from performing the index test or the reference standard	na
**DISCUSSION**	26	Study limitations, including sources of potential bias, statistical uncertainty, and generalisability	Yes
	27	Implications for practice, including the intended use and clinical role of the index test	Yes (novel TNM-Immune classification for Stage I/III colon cancer)
**OTHER INFORMATION**	28	Registration number and name of registry	na
	29	Where the full study protocol can be accessed	Yes
	30	Sources of funding and other support; role of funders	Yes

Table S5: STARD checklist

Forest Plot of Effect of Immunoscore Int+Hi (25-100%) vs Lo (0-25%) on Time To Recurrence (TTR) Stage I-III Patients

Subgroup	Events/ Total	Immunoscore 0−25%/ 25−100%	Multivariable Hazard Ratio (95% CI)*	Wald	Interaction P Value**	Association P Value***
Overall	65/423	158/265	0.53 (0.32−0.86)	0.0097		
Age at surgery (years)					0.7544	0.3481
<70	42/268	105/163	0.56 (0.31−1.03)	0.0622		
>=70	23/155	53/102	0.47 (0.21−1.07)	0.072		
Gender					0.5214	0.4198
Male	34/231	82/149	0.45 (0.23−0.89)	0.0221		
Female	31/192	76/116	0.61 (0.3−1.24)	0.1738		
T Stage					0.6235	0.0093
T1−2	3/73	16/57	Inf (0−Inf)	0.9991		
T3	45/289	118/171	0.52 (0.29−0.94)	0.0299		
T4	17/61	24/37	0.55 (0.21−1.44)	0.2245		
N Stage					0.0474	0.594
N0	35/318	117/201	0.35 (0.17−0.69)	0.0025		
N1	12/66	28/38	0.73 (0.23−2.25)	0.578		
N2	18/39	13/26	0.93 (0.36−2.4)	0.8733		
Post-Operative Chemotherapy					0.4833	0.6722
NO	15/146	53/93	0.3 (0.1−0.87)	0.0262		
YES	47/265	102/163	0.62 (0.35−1.1)	0.1028		
Proximal vs. Distal Primary (Tumor)					0.9343	0.1849
Proximal	26/184	62/122	0.47 (0.22−1.01)	0.0543		
Distal	38/232	94/138	0.61 (0.32−1.16)	0.1339		
Very High Risk (T4 and VELIPI+)					0.443	0.6872
Very High Risk	6/27	11/16	0.32 (0.06−1.72)	0.1828		
Low Risk	59/396	147/249	0.56 (0.33−0.93)	0.0248		
High Risk (T4 or VELIPI+)					0.7787	0.0874
High Risk	56/311	124/187	0.52 (0.31−0.88)	0.0142		
Low Risk	9/112	34/78	0.64 (0.17−2.38)	0.5053		

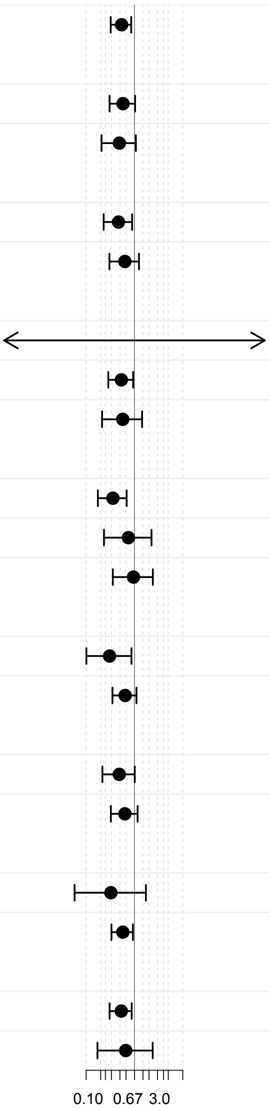

* Subgroup Analysis for Immunoscore with Lo (0-25%) as ref.
** Interaction Wald PValue from interaction term parameter:group.
***Association PValue from two-sided Fisher's exact test.

Figure S1

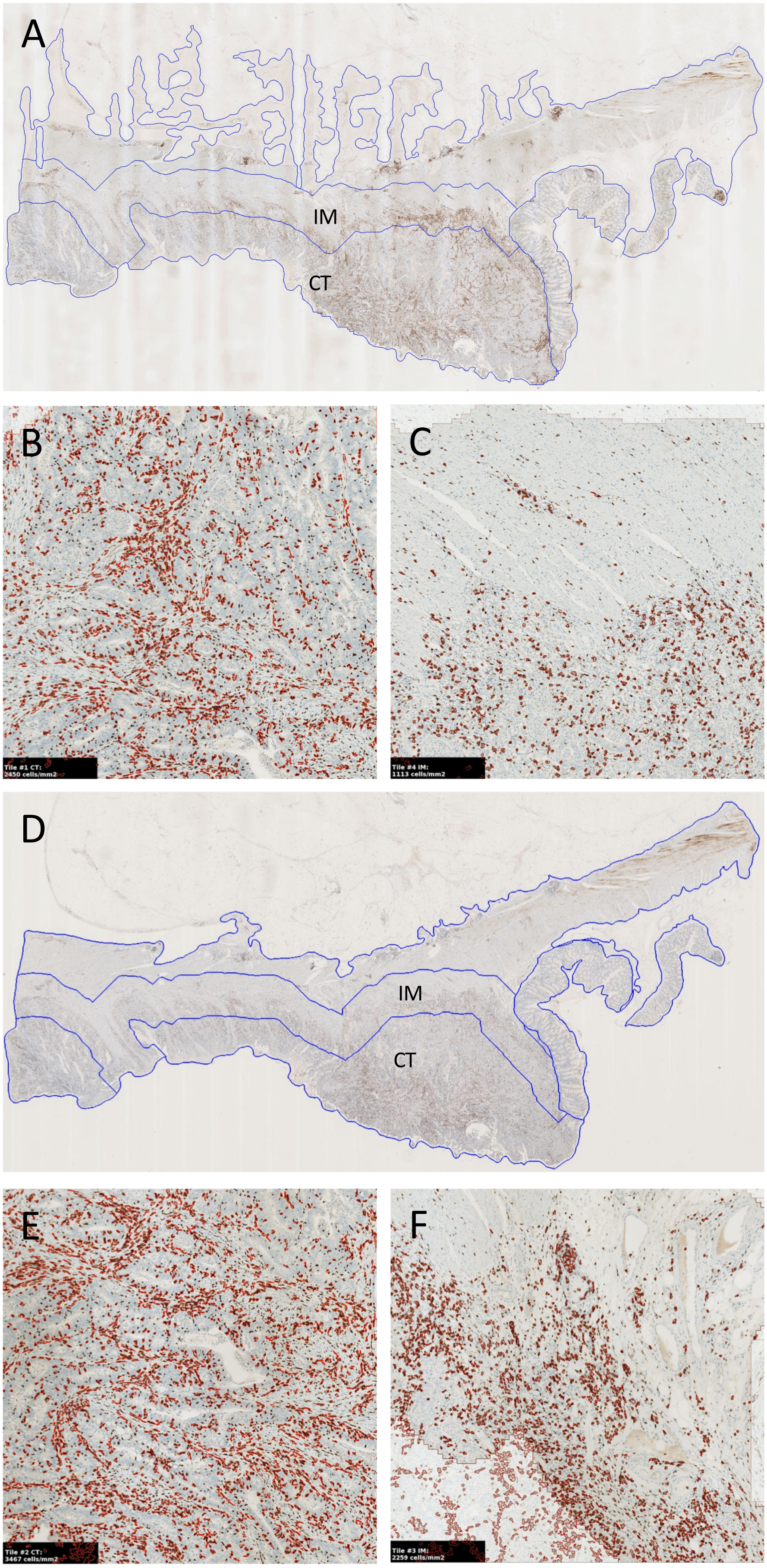

Figure S2: CD8 staining (A, B, C) and CD3 staining (D, E, F) of a patient from Japan, low magnification of the whole slide with CT and IM regions (A, D),and high magnification of CT (B, E) and IM (C, F) regions.

Figure S2
